# Characterization of resistance and fitness cost of *Descurainia sophia* L. populations from Henan and Xinjiang, China

**DOI:** 10.1038/s41598-021-94317-y

**Published:** 2021-07-19

**Authors:** Dongzhi Li, Lanfen Xie, Pei Zhang, Runqiang Liu, Mingwang Shi, Yu Mei, Li Xu

**Affiliations:** 1 College of Resources and Environment, Henan Institute of Science and Technology, Hualan Road, Hongqi District, Xinxiang, 453003 Henan Province China; 2 State Key Laboratory of Desert and Oasis Ecology, Xinjiang Institute of Ecology and Geography, Chinese Academy of Sciences, Beijing South Road, Xinshi District, Urumqi, 830011 China; 3 The Specimen Museum of Xinjiang Institute of Ecology and Geography, Chinese Academy of Sciences, Urumqi, 830011 China

**Keywords:** Ecology, Plant sciences

## Abstract

*Descurainia sophia* L. is a notorious weed in winter wheat field and has serious resistance to tribenuron-methyl. Xinjiang is a main wheat production region in China with no information on *D. sophia* resistance to tribenuron-methyl. Here, resistance levels of *D. sophia* populations to tribenuron-methyl from Xinjiang and Henan were investigated. In addition, homozygous mutation subpopulations of high resistant *D. sophia* populations from Xinjiang and Henan were generated and then cross-resistance and fitness cost were determined. Results showed that 5 out of 31 populations from Xinjiang developed resistance to tribenuron-methyl, including two high resistant populations (X30 and X31). While 10 out of 11 populations from Henan showed resistance to tribenuron-methyl, including three high resistant populations (H5, H6 and H7). X30 and X31 shared the same mutation type of Pro197Thr in *ALS1*, while the mutation type of *ALS1* in H5, H6 and H7 were Pro197Ser, Pro197His and Pro197Ala, respectively. The homozygous mutation subpopulations (SX30, SX31, SH5, SH6, SH7) showed cross-resistance to flucarbazone-sodium, bensulfuron methyl and flumetsulam. Under monoculture condition, relative growth rates of SX30, SX31 were higher than susceptible population (SX13), while that in SH5, SH6, SH7 were almost same with SX13. When mix planted with SX13, SX30 and SX31 displayed weaker competitiveness than SX13, while SH5, SH6, SH7 showed stronger competitiveness than SX13. The results suggested that *D. sophia* from Xinjiang had low resistance frequency to tribenuron-methyl and the high resistant populations had fitness costs.

## Introduction

*Descurainia sophia* L. is a malignant broad-leaf weed infesting winter wheat and has developed serious resistance to tribenuron-methyl in China^1–4^. Tribenuron-methyl at recommended dose cannot control *D. sophia* effectively, which affect the yield and quality of winter wheat seriously. It has been reported that the high tribenuron-methyl resistance level in *D. sophia* is mainly conferred by target-site-based resistance (TSR) caused by Pro197 (substituted by Ala, Arg, Leu, Thr, Ser and His) or Asp376 (by Glu) or Trp574 (by Leu) in acetolactate synthase (ALS) and non-target-site-based resistance (NTSR) caused by cytochrome P450^1–3,5–8^, and its occurrence was often accompanied with fitness cost.

Fitness is a key factor regulating weed resistance evolution. Fitness cost developed due to the herbicide resistance weed needs extra energy to maintain the normal function of the mutant target protein^9,10^. Low fitness cost indicates strong competition ability and fast spread of resistant population^11^, while high fitness cost suggests weak competition ability and slow spread of resistant population^12,13^. Under the same growth condition, different target site mutation in TSR always cause different fitness cost^14–16^. Pro197Arg mutation decreased the growth rate in *Lolium rigidum*, while no fitness cost was observed in populations with Pro197Ser and Trp574Leu mutations^14^. It was reported that the resistant *D. sophia* plants collected from Hebei Province carrying Pro197Leu or Pro197His or Asp376Glu or Trp574Leu showed stronger competitiveness than susceptible plants without mutation and no obviously negative effects were detected on the pigments, relative growth rates (RGR), leaf area ratio (LAR) and net assimilation rate (NAR) in resistant plants^17^.

Henan and Xinjiang are the main production regions of winter wheat in north and northwest of China, respectively. *D. sophia* is the well-known dominant species in the winter wheat field of Henan^18^. Xinjiang is a sub-region in the national wheat planting area because of its unique geographical location and climatic environment, and it is also the only wheat planting area divided by a single province or autonomous region in China^19^. *D. sophia* was the absolute dominant species in winter wheat planting area of Xinjiang, especially in Kashgar, where *D. sophia* accounted for 50% of total weed^20,21^. However, information on the resistance and fitness cost of *D. sophia* from Xinjiang is still limited. Considering the risk of *D. sophia*, this study focused on the following objectives: (1) determine the resistance level and mechanism of *D. sophia* from Henan and Xinjiang to tribenuron-methyl; (2) investigate cross-resistance to other ALS-inhibiting herbicides; (3) evaluate the fitness cost of different *D. sophia* populations from Henan and Xinjiang.

## Results

### Tribenuron-methyl dose response

**Figure 1:**
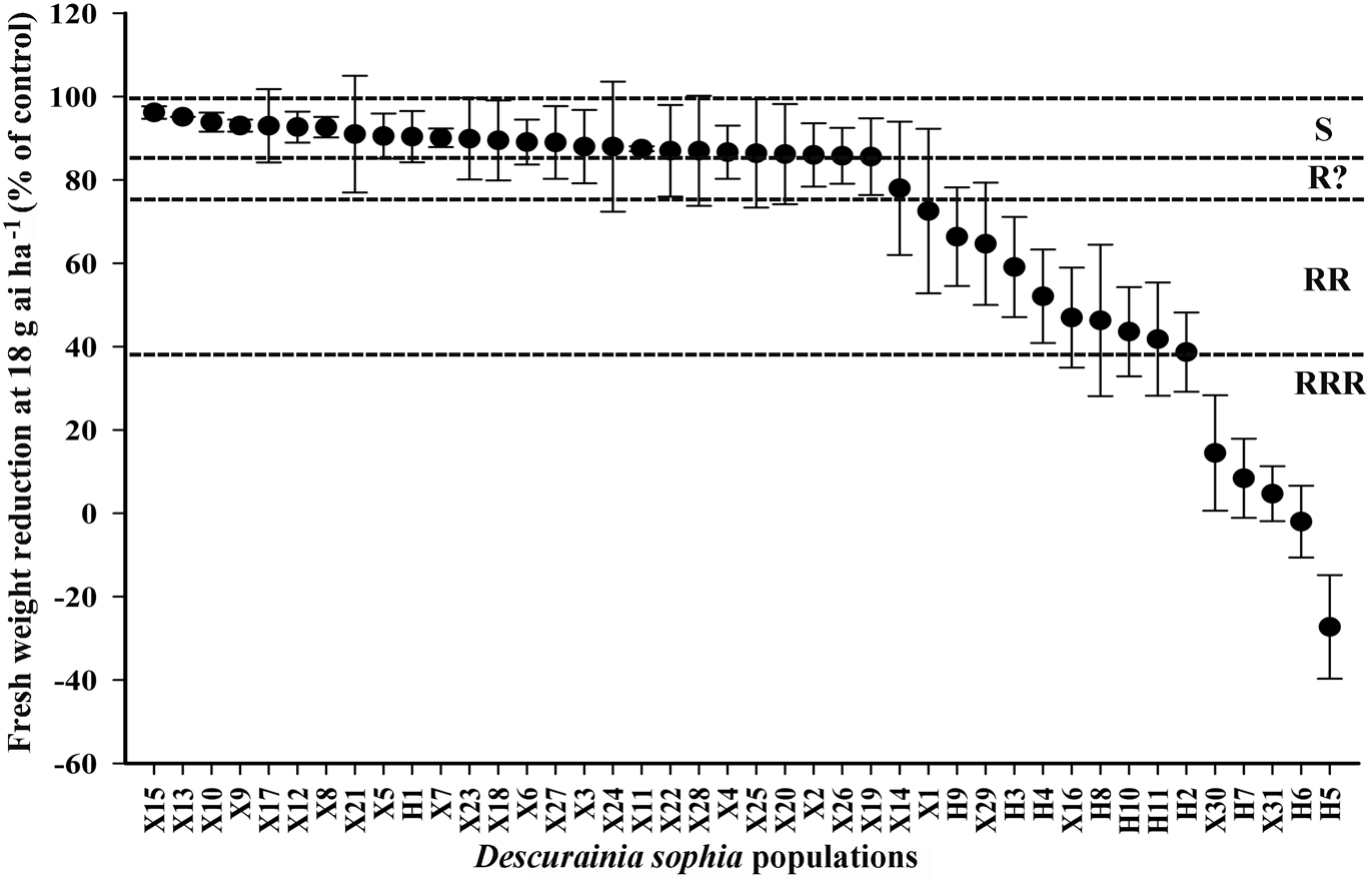
The fresh weight reduction rate of 42 *D. sophia* populations under the discriminating dose of tribenuron-methyl at 18 g ai ha^−1^ from Henan and Xinjiang. According to “R” resistance rating system, the *D. sophia* populations were divided into: (1) RRR (high resistance), fresh weight reduction between 0 and 38.1%; (2) RR (resistance), fresh weight reduction between 38.1 and 76.1%; (3) R? (potential resistance), fresh weight reduction between 76.1 and 85.6%; (4) S (susceptible), fresh weight reduction between 85.6 and 100%; Each point represents the mean ± SD of three replications.

**Figure 2:**
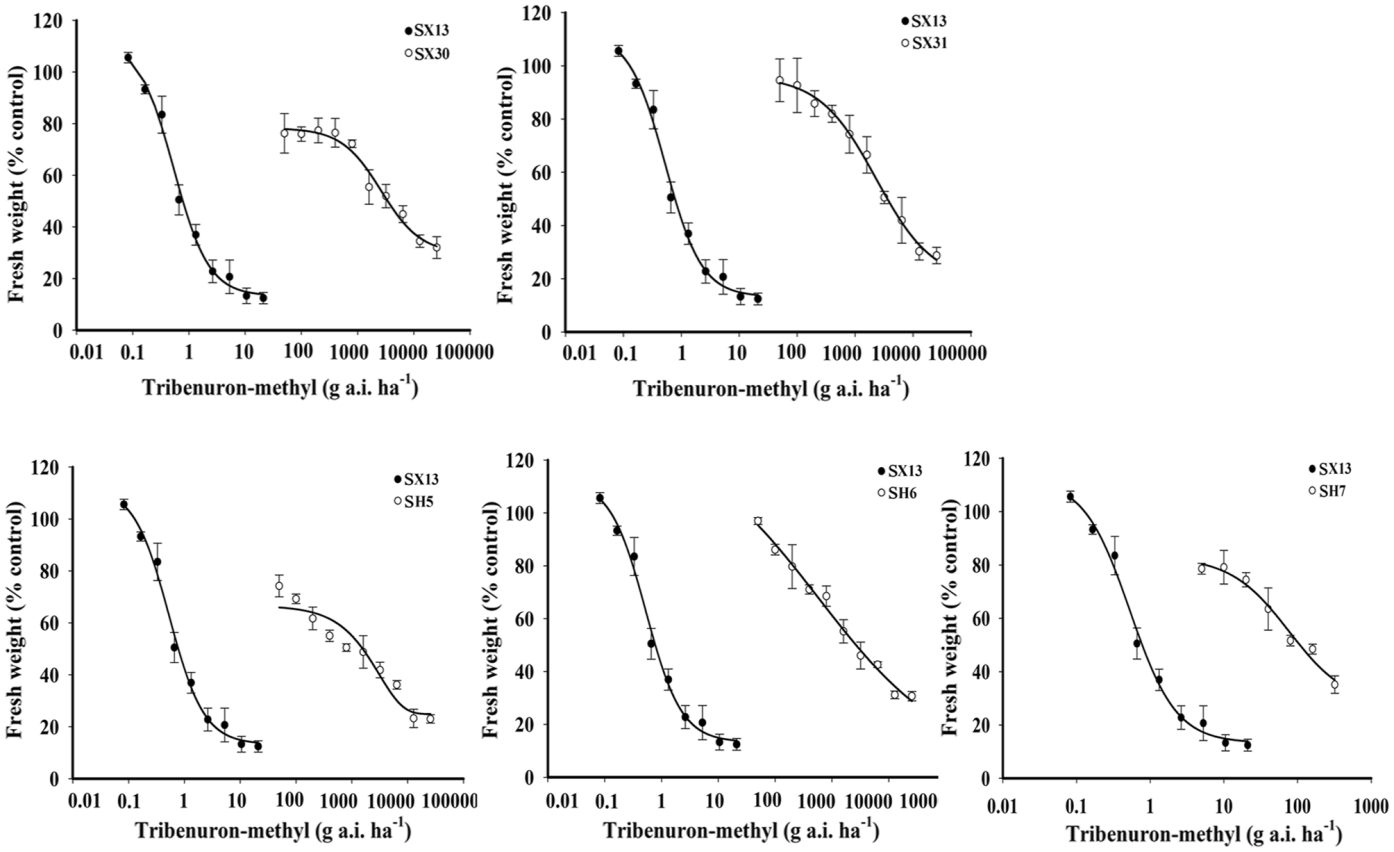
Dose response of high resistance *D. sophia* subpopulations to tribenuron-methyl.

The bioassay results of single dose resistance assay showed that 5 out of 31 *D. sophia* populations from Xinjiang, namely X1, X16, X29, X30, X31, developed resistance to tribenuron-methyl. A total of 10 populations from Henan (H2, H3, H4, H5, H6, H7, H8, H9, H10, H11) was determined to develop resistance to tribenuron-methyl (Fig. 1). X30, X31 from Xinjiang, and H5, H6, H7 from Henan were identified to have high resistance (RRR) to tribenuron-methyl evaluated by Moss et al.^23^ The GR_50_ values of purified homozygous subpopulations SX30, SX31, SH5, SH6, SH7 were 2633 ± 685, 2326 ± 415, 1547 ± 332, 576 ± 64, 77 ± 22 g ai ha^−1^, respectively. Compared with the GR_50_ of SX13 (0.53 ± 0.08 g ai ha^−1^), the resistance index (RI) of SX30 and SX31 were 4968- and 4389-fold, while the RI of SH5, SH6 and SH7 were 2919-, 1087- and 145-fold, respectively (Fig. 2).

### Detection of target-site mutation

**Figure 3:**
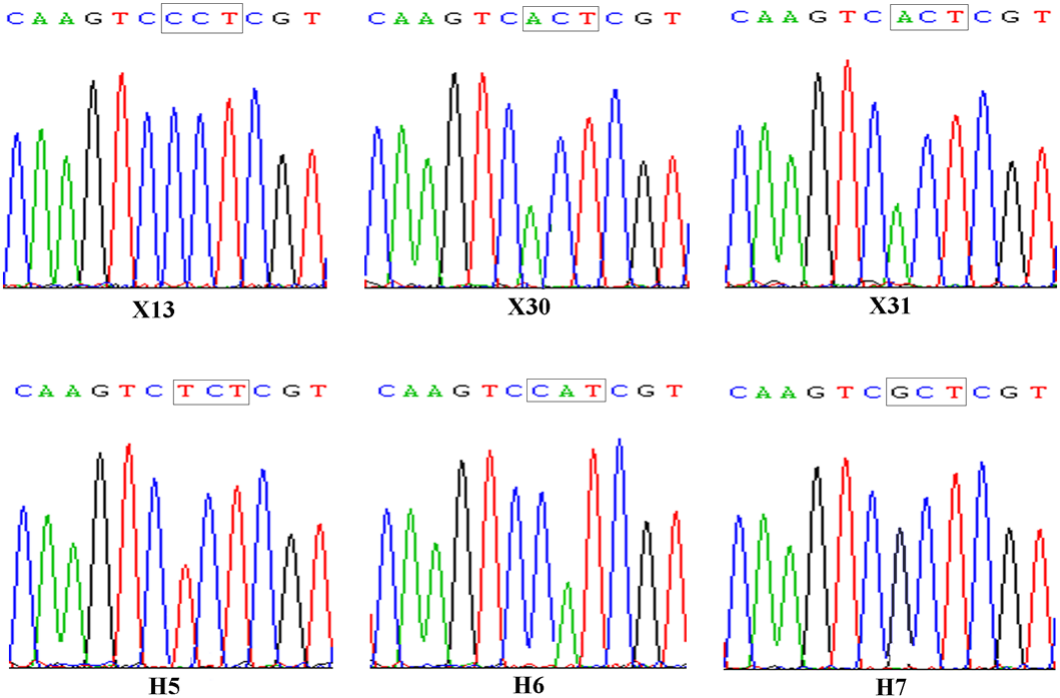
Mutation type of Pro197 in *ALS1* of the high resistant populations of *D. sophia*.

The mutation type of ALS isozymes in the five high resistant *D. sophia* populations (SX30, SX31, SH5, SH6, SH7) were detected. According to sequence alignment, only *ALS1* and *ALS2* were identified in the RRR populations of *D. sophia*. No mutation was found in the susceptible population X13. For *ALS1*, Pro197Thr was detected in X30 and X31, while Pro197Ser in H5, Pro197His in H6 and Pro197Ala in H7 (Fig. 3). There was no mutation in *ALS2* among all of the detected populations.

### Cross-resistance to other ALS-inhibiting herbicides of RRR subpopulations of *D. sophia* with different mutation type

The cross-resistance to other ALS-inhibiting herbicides of RRR subpopulations of *D. sophia* with different mutation were evaluated. Results showed that SX30 with Pro197Thr mutation had cross-resistance to flucarbazone-sodium (sulfonylamino-carbonyl-triazolinone, SCT), bensulfuron methyl (sulfonylurea, SU), flumetsulam (triazolopyrimidine, TP), pyroxsulam (TP). SX31 with Pro197Thr mutation had cross-resistance to flucarbazone-sodium (SCT), bensulfuron methyl (SU), flumetsulam (TP), pyroxsulam (TP), imazapic (imidazolinone, IMI) and bispyribac-sodium (pyrimidinyl-thiobenzoat, PTB). SH5 with Pro197Ser mutation developed cross-resistance to flucarbazone-sodium (SCT), bensulfuron-methyl (SU) and flumetsulam (TP). SH6 with Pro197His mutation developed cross-resistance to flucarbazone-sodium (SCT), bensulfuron-methyl (SU), flumetsulam (TP), florasulam (TP) and bispyribac-sodium (PTB). SH7 with Pro197Ala mutation developed cross-resistance to all the tested ALS-inhibiting herbicides (Table 1).

**Table 1:** The cross-resistance to other ALS-inhibition herbicides of the high resistant subpopulations of *D. sophia*.

Purified subpopulation	ALS mutation		Plants with specific ALS mutations survived in 1×, 5× and 10× fold recommended dose (%)																					
	ALS1	ALS2	Flucarbazone-sodium (45.0 g ai ha^−1a^, SCT)		Bensulfuron methyl (65.7 g ai ha^−1a^, SU)					Flumetsulam (30.0 g ai ha^−1a^, TP)			Florasulam (4.5 g ai ha^−1a^, TP)			Pyroxsulam (14.1 g ai ha^−1a^, TP)			Imazapic (108.0 g ai ha^−1a^, IMI)			Bispyribac-sodium (45.0 g ai ha^−1a^, PTB)		
			1×	5×		10×	1×	5×	10×	1×	5×	10×	1×	5×	10×	1×	5×	10×	1×	5×	10×	1×	5×	10×
SX13	Wild type	Wild type	30	0		0	0	0	0	0	0	0	0	0	0	0	0	0	0	0	0	0	0	0
SX30	Pro197Thr	Wild type	100	100		100	100	100	100	100	100	100	0	0	0	60	0	0	0	0	0	0	0	0
SX31	Pro197Thr	Wild type	100	100		100	100	100	100	100	100	100	0	0	0	100	100	100	100	50	50	100	0	0
SH5	Pro197Ser	Wild type	100	100		100	100	100	100	100	100	100	20	0	0	0	0	0	0	0	0	0	0	0
SH6	Pro197His	Wild type	100	100		100	100	100	100	100	100	100	100	50	30	30	0	0	30	0	0	100	0	0
SH7	Pro197Ala	Wild type	100	100		100	100	100	100	100	100	100	100	0	0	100	50	0	100	20	0	50	0	0

*SCT* sulfonylamino-carbonyl-triazolinones, *SU* sulfonylurea, *TP* triazolopyrimidine, *IMI* imidazolinone, *PTB* pyrimidinyl-thiobenzoate.

^a^The onefold discriminating-dose known to control the S population.

### The fitness cost of RRR subpopulations of *D. sophia*

Under monoculture, the leaf area and dry weight of individual plant with different mutation were listed in Table S3. Compared with the susceptible subpopulation SX13, SX30 and SX31 with Pro197Thr mutation showed significantly higher leaf area and dry weight at 42 DAT. The RGR and NAR in SX30 and SX31 during 28–42 DAT were also significantly higher than SX13 (Tables 2 and 3). No significant difference on LAR were observed between the susceptible and resistant subpopulations (Table 4). However, the effects of resistance mutation on nutritional growth level of SH5, SH6 and SH7 were complex. For example, SH5 with Pro197Ser mutation had significantly smaller leaf area at 42 DAT and significantly higher NAR during 28–42 DAT compared with SX13. SH6 with Pro197His mutation had significantly smaller leaf area and dry weight at 35 DAT. RGR and NAR in SH6 were significantly lower during 28–35 DAT, but significantly higher during 35–42 DAT. SH7 with Pro197Ala mutation had significantly higher leaf area at 42 DAT, but significantly lower RGR and NAR during 28–35 DAT.

**Table 2:** The RGR of *D. sophia* subpopulations from Henan and Xinjiang under monoculture condition.

DAT	RGR (mg mg^−1^ day^−1^)					
	SX13	SX30	SX31	SH5	SH6	SH7
28–35	0.118 ± 0.023b	0.077 ± 0.012a	0.190 ± 0.024c	0.120 ± 0.015b	0.078 ± 0.011a	0.073 ± 0.012a
35–42	0.123 ± 0.018ab	0.268 ± 0.033d	0.142 ± 0.026b	0.104 ± 0.014a	0.200 ± 0.019c	0.150 ± 0.016b
28–42	0.121 ± 0.016a	0.172 ± 0.019b	0.166 ± 0.031b	0.112 ± 0.017a	0.140 ± 0.020ab	0.112 ± 0.015a

Different lowercase indicated significant difference with p <0.05.

**Table 3:** The NAR of *D. sophia* subpopulations from Henan and Xinjiang under monoculture condition.

DAT	NAR (mg cm^−2^ day^−1^)						
	SX13	SX30	SX31		SH5	SH6	SH7
28–35	0.377 ± 0.042c	0.371 ± 0.034c		0.432 ± 0.035c	0.329 ± 0.027bc	0.112 ± 0.009a	0.292 ± 0.032b
35–42	0.309 ± 0.028a	0.679 ± 0.082c		0.336 ± 0.029a	0.880 ± 0.102d	0.445 ± 0.056b	0.289 ± 0.037a
28–42	0.323 ± 0.026b	0.451 ± 0.052cd		0.389 ± 0.041c	0.601 ± 0.065d	0.289 ± 0.017ab	0.236 ± 0.019a

Different lowercase indicated significant difference with p <0.05.

**Table 4:** The LAR of *D. sophia* subpopulations from Henan and Xinjiang under monoculture condition.

DAT	LAR (cm^2^ mg^−1^)					
	SX13	SX30	SX31	SH5	SH6	SH7
28–35	0.521 ± 0.087a	0.638 ± 0.096a	0.606 ± 0.096a	0.528 ± 0.093a	0.647 ± 0.088a	0.603 ± 0.052a
35–42	0.433 ± 0.082ab	0.457 ± 0.065ab	0.486 ± 0.068b	0.352 ± 0.067a	0.560 ± 0.064b	0.523 ± 0.046b
28–42	0.489 ± 0.079ab	0.513 ± 0.069b	0.543 ± 0.076b	0.379 ± 0.061a	0.547 ± 0.049b	0.586 ± 0.056b

Different lowercase indicated significant difference with p <0.05.

The relative competition ability of susceptible and resistant subpopulations was evaluated under five different mix planting proportions (1:0, 3:1, 1:1, 1:3 and 0:1). Results showed that under the competition environment, relative crowding coefficient (RCC) of dry weight and leaf area in SX30, SX31, SH5, SH6, SH7 were 1.11, 1.09, 0.80, 0.79, 0.85 and 1.45, 1.12, 0.58, 0.91, 0.97, respectively, (Tables 5 and 6).

**Table 5:** The dry weight and RCC of *D. sophia* subpopulations from Henan and Xinjiang under admixture condition.

Population	Biotype	Dry weight of different mix plant ratio (S:R) (mg)					RCC
		1:0	3:1	1:1	1:3	0:1	
SX30	S	53.5 ± 6.1a	66.4 ± 7.2a	61.5 ± 7.2a	52.8 ± 6.3a	0	1.11
	R	0	87.3 ± 6.2b	57.7 ± 6.2a	67.1 ± 4.3a	69.3 ± 7.1a	
SX31	S	53.5 ± 6.1ab	47.6 ± 3.9a	61.3 ± 5.8b	50.7 ± 4.9ab	0	1.09
	R	0	50.5 ± 6.2a	87.8 ± 9.6c	57.6 ± 5.1a	70.4 ± 6.3b	
SH5	S	53.5 ± 6.1a	46.9 ± 5.6a	51.8 ± 4.1a	50.3 ± 4.7a	0	0.80
	R	0	36.8 ± 4.6a	60.8 ± 5.8c	51.7 ± 6.5bc	50.6 ± 4.5b	
SH6	S	53.5 ± 6.1a	60.7 ± 5.7a	51.7 ± 5.3a	61.0 ± 6.2a	0	0.79
	R	0	92.3 ± 8.8c	34.5 ± 3.7a	69.1 ± 7.8b	41.9 ± 3.5a	
SH7	S	53.5 ± 6.1b	53.69 ± 4.5b	37.0 ± 4.3a	83.3 ± 7.6c	0	0.85
	R	0	103.2 ± 9.1b	60.8 ± 5.2a	71.4 ± 8.2a	59.7 ± 5.9a	

Different lowercase indicated significant difference with p <0.05.

**Table 6:** The leaf area and RCC of *D. sophia* subpopulations from Henan and Xinjiang under admixture condition.

Population	Biotype	Leaf area of different mix plant ratio (S:R) (cm^2^)					RCC
		1:0	3:1	1:1	1:3	0:1	
SX30	S	23.21 ± 2.13a	34.54 ± 3.14c	30.32 ± 2.36bc	25.65 ± 2.81ab	0	1.45
	R	0	35.61 ± 2.65b	30.87 ± 2.31ab	32.22 ± 3.12ab	29.52 ± 1.87a	
SX31	S	23.21 ± 2.13ab	19.83 ± 2.17a	25.84 ± 1.98b	20.52 ± 1.68a	0	1.12
	R	0	20.03 ± 2.12a	42.15 ± 1.87c	24.80 ± 2.51a	32.1 ± 2.32b	
SH5	S	23.21 ± 2.13b	13.98 ± 2.38a	23.88 ± 2.62b	22.02 ± 2.51b	0	0.58
	R	0	16.74 ± 2.57a	24.75 ± 2.16b	23.08 ± 3.16b	14.6 ± 1.36a	
SH6	S	23.21 ± 2.13ab	28.56 ± 2.35b	21.51 ± 2.12a	23.46 ± 2.57ab	0	0.91
	R	0	41.32 ± 3.25d	15.63 ± 1.69a	27.69 ± 3.15c	21.7 ± 1.58b	
SH7	S	23.21 ± 2.13a	23.30 ± 2.63a	18.82 ± 2.18a	36.76 ± 3.69b	0	0.97
	R	0	46.80 ± 2.57b	31.67 ± 3.26a	33.71 ± 3.51a	31.0 ± 1.98a	

Different lowercase indicated significant difference with p <0.05.

## Discussion

*Descurainia sophia* resistance to tribenuron-methyl was widely reported in the winter wheat planted area of northern China, including Henan, Shandong, Hebei, Tianjin, Shanxi, Shaanxi, Jiangsu Province^1–4^. Xinjiang is also a main planted area of winter wheat and its yield accounted for 2.95% of the whole production of winter wheat in China in 2018 (http://zdscxx.moa.gov.cn:8080/nyb/pc/search.jsp). Xinjiang is divided as northern and southern Xinjiang. Southern Xinjiang is mainly planted with winter wheat, while northern Xinjiang is mainly planted with spring wheat and adopted mono-cropping system^21^.

Our results showed that X1, X16 from northern Xinjiang and X29 from southern Xinjiang developed resistance to tribenuron-methyl, while X30, X31 from southern Xinjiang (Kashgar) developed high resistance to tribenuron-methyl, which indicated that *D. sophia* resistance to tribenuron-methyl had emerged in both northern and southern Xinjiang and more severe in southern Xinjiang. This might be related with the different planting structure between northern and southern Xinjiang. 10 out of 11 *D. sophia* populations collected from Henan were determined to have resistance to tribenuron-methyl and three out of ten populations were determined to have high resistance to tribenuron-methyl. The resistant frequency of *D. sophia* populations from Xinjiang is only 16.1%, which is much lower than Henan (90.9%) and Hebei, Shandong, Shanxi, Shaanxi, Jiangsu reported in Xu et al.^1^.

Results in TSR detection suggested that X30 and X31 from Xinjiang shared the same mutation type of Pro197Thr, and H5, H6, H7 from Henan had three mutation types, which indicated that the mutation type in high resistant populations from Xinjiang was less various than Henan. All the five high resistant subpopulations showed cross-resistance to flucarbazone-sodium (SCT), bensulfuron methyl (SU), flumetsulam (TP), which was similar with previous report^6^. SX30 and SX31 with same mutation type displayed different herbicide cross-resistance pattern, which might result from different resistance mechanism caused by genetic background variance between SX30 and SX31. SH7 with Pro197Ala mutation showed cross-resistance to all seven ALS-inhibiting herbicides, which indicated that the resistance in SH7 was more difficult to deal with.

Under monoculture, RGR in SX30 and SX31 were higher than the susceptible population during the whole detected period, while that in SH5, SH6, SH7 were almost the same with the susceptible population. LAR in all the high resistant subpopulations showed no significant difference with susceptible population, which was similar with the founding in Zhang et al.^17^. Under admixture condition, SX30 and SX31 displayed lower competition ability compared to the susceptible population for their RCC of both leaf area and dry weight higher than 1, while SH5, SH6, SH7 displayed higher competition ability for their RCC lower than 1. RCC of leaf area and dry weight in populations with Pro197His and Pro197Ser mutation were lower than 1 in this work and Zhang et al.^17^, and RCC of leaf area in populations with Pro197Thr mutation was higher than 1 in this work and Zhang et al.^17^. Thus, it was speculated that SX30 and SX31 with Pro197Thr mutation from Xinjiang had fitness cost, while SH5 and SH6 with Pro197His and Pro197Ser mutation from Henan had no obvious fitness cost. Besides, SH7 with Pro197Ala mutation from Henan was suggested to have no obvious fitness cost, either.

Fitness cost is the combination result caused by genetic background, resistance mechanisms, specific resistance alleles, characteristics of target enzyme, weed species and growth environment^22^. Generally, genetic background has great influence on fitness cost^14^, it is necessary to manage the genetic differences between populations that are not associated with resistance mutation. There are several ways to do this, such as creating near-isogenic lines or using F2 segregating populations. Here, we focused on the fitness cost of populations from two geographical locations instead of different mutation types. This kind of fitness cost comparison was meaningful on the resistance risk evaluation between Henan and Xinjiang. Our work suggested that subpopulations with high tribenuron-methyl resistance from Henan (SH5, SH6, SH7) had strong competition ability, and their resistance was easy to spread. When introduced winter wheat seeds from other provinces to Xinjiang, more attention should be paid on the seed quality and the mixture of *D. sophia* seeds must be avoided.

Overall, *D. sophia* populations from Xinjiang had developed resistance to tribenuron-methyl, and the resistance in southern Xinjiang was much severer than northern Xinjiang. The resistance frequency in Xinjiang was much lower than Henan. The mutation type of Pro197 in Xinjiang was less various than Henan. Flucarbazone-sodium (SCT), bensulfuron methyl (SU) and flumetsulam (TP) were not recommended to substitute tribenuron-methyl to control *D. sophia* for its common cross-resistance in all the high resistant populations. The resistance risk of *D. sophia* to tribenuron-methyl in Xinjiang was relatively low, and it is important to control the import of *D. sophia* seeds from other provinces. These results provide valuable basis for the scientific evaluation of resistance development in Xinjiang and give a new idea for its ecological management.

## Materials and methods

### Plant material

A total of 42 *D. sophia* populations were collected from winter wheat fields of Henan (H1-11) and Xinjiang (X1-31) in China during 2015–2017. The geographical origin and collection year of *D. sophia* populations were provided in Table S1 and Fig. S1. A tribenuron-methyl susceptible population of *D. sophia* (X13) was collected from Urumqi, Xingjiang.

Seeds were soaked with 15% H_2_O_2_ for 30 min to break dormancy and rinsed thoroughly by water. The treated seeds were placed in moist petri dishes and then transferred to artificial climate chamber at 15 ℃, light/dark, 16/8 h for germination. After a week, the seedlings were transplanted to 10-cm diameter plastic pots containing loam soil (12 plants per pot) and then cultured in artificial climate chamber at 25/15 ℃, light/dark, 16/8 h with light intensity of 15,000 Lux. The seedlings were used in the following procedures.

### Single dose resistance assay

The discriminating dose of tribenuron-methyl at 18 g ai ha^−1^ was sprayed to the plants at 4-leaf stage using a potter precision laboratory spray tower (Burkard Scientific, UK) delivering 600 L ha^−1^ water at the pressure of 0.3 MPa. The fresh weight of aboveground of the plants were determined after tribenuron-methyl application for 21 days and the fresh weight reduction rate were calculated. The susceptibility of *D. Sophia* populations to tribenuron-methy was identified according to Moss et al.^23^ and populations classified as high resistance (RRR) were selected for further mutation type determination.

### Detection of ALS isozymes mutation

Genomic DNA of RRR *D. sophia* populations were extracted from the survived plant using Wizard^®^ Genomic DNA Purification Kit (Promega, Madison, WI). Primer pairs, PCR reaction and program cycle in Xu et al.^1^ were used to detect the eight resistance mutation sites in ALS isozymes. PCR products were purified with Wizard^®^ SV Gel and PCR Clean-Up System (Promega) and inserted to pLB vector using Lethal Based Fast Cloning Kit (Tiangen, Beijing, China). The mixture was transformed to TOP10 competent *E. coli* (Tiangen) and finally sequenced by Shanghai Sangon Biological Engineering and Technology Service Co. (Shanghai, China). Ten individual plants and three clones of each were selected for *ALS* mutation detection.

### Generation of RRR homozygous subpopulations

Plants of RRR population with same mutation type in ALS isozymes were cultured to generate seeds. Homozygous subpopulation of susceptible population X13 with wild type of ALS isozymes were also obtained by inbred. In this way, six purified subpopulations (SX13, SX30, SX31, SH5, SH6, SH7) homozygous for wild type, Pro197Ser, Pro197His, Pro197Ala, Pro197Thr mutations, were obtained and used for the following experiments.

### Dose response of RRR *D. sophia* subpopulations to tribenuron-methyl

Whole-plant dose response experiment was employed to identify the GR_50_ of the RRR homozygous subpopulations. Seeds of subpopulation were cultured as mentioned previously. Tribenuron-methyl was applied to SX13 (0, 0.08, 0.16, 0.33, 0.66, 1.32, 2.64, 5.28, 10.56, 21.12 g ai ha^−1^), SH7 (0, 5, 10, 20, 40, 80, 160, 320 g ai ha^−1^) and SH5, SH6, SX30, SX31 (0, 50, 100, 200, 400, 800, 1600, 3200, 6400, 12,800, 25,600 g ai ha^−1^) subpopulations at 4-leaf stage using a potter precision laboratory spray tower delivering 600 L ha^−1^ water at the pressure of 0.3 MPa. The aboveground of the plants were harvested after treated for 21 days and the fresh weight was recorded. Each herbicide dose was conducted with three replications and repeated twice. GR_50_ was calculated by log-logistic equation^24^:

$${\text{y}} = {\text{C}} + {{\left( {{\text{D}} - {\text{C}}} \right)} \mathord{\left/ {\vphantom {{\left( {{\text{D}} - {\text{C}}} \right)} {\left[ {1 + \left( {{\text{x}}/{\text{GR}}_{50} } \right)^{{\text{b}}} } \right]}}} \right. \kern-\nulldelimiterspace} {\left[ {1 + \left( {{\text{x}}/{\text{GR}}_{50} } \right)^{{\text{b}}} } \right]}}$$

where C and D are the lower limit and upper limit, b is the slope, x is the herbicide dose, and y represents plant fresh weight as percentage of the control. RI, the ratio of GR_50_ of resistant populations to that of the susceptible population, was used to represent the resistance level.

### Cross-resistance patterns of RRR *D. sophia* subpopulations to other ALS-inhibiting herbicides

Other ALS-inhibiting herbicides, including flucarbazone-sodium (SCT), bensulfuron-methyl (SU), flumetsulam (TP), florasulam (TP), pyroxsulam (TP), imazapic (IMI) and bispyribac-sodium (PTB) were applied to *D. sophia* at 4-leaf stage with 1×, 5× and 10× fold of the recommendation doses. The herbicides and recommendation doses were listed in Table S2. The survival plant was recorded after treated for 21 days and each dose was replicated with three plastic pots containing 36 plants. Cross-resistance was confirmed as more than 50% individuals survived in the resistant population and less than 10% plants survived in the susceptible population^6,25^.

### Determination of RGR, LAR, NAR and RCC in susceptible and RRR subpopulations

RGR, LAR and NAR were used to indicate the nutritional growth level of susceptible and resistant homozygous subpopulations of *D. sophia*. RCC was used to evaluate their relative competition ability. RGR, LAR, NAR and RCC were determined according to Zhang et al. with a little modification^17^.

Under monoculture condition, seeds of each subpopulation were planted separately with three replications and repeat twice. The aboveground tissues of *D. sophia* without herbicide treatment were sampled at 28, 35 and 42 days after transplant (DAT) to compare the nutritional growth between susceptible and resistant subpopulations. All leaves of the harvested plant were placed on A4 paper drawing with 1 cm^2^ square and photoed to calculate the leaf area by Photoshop CS3 extended (Adobe Systems Inc., USA). The dry weight was measured after the sample oven dried 96 h at the temperature of 60 ℃. RGR was estimated by the formula RGR = (ln W2 − ln W1)/(t2 − t1)^26^. LAR and NAR were calculated by the formula LAR = [(ln W2 − ln W1)(LA2 − LA1)]/[(W2 − W1)(ln LA2 − ln LA1)] and NAR = [(W2 − W1) (ln W2 − ln W1)]/[(LA2 − LA1)(t2 − t1)]^27^. W1 and W2 indicated dry weight per plant at times t1 and t2, respectively. LA1 and LA2 means leaf area per plant at t1 and t2, respectively.

Under admixture condition, plants of susceptible and resistant subpopulations were cultured at a series ratio of S:R = 1:0, 3:1, 1:1, 1:3, 0:1 at a constant density of 644 plants m^−2^ (24 plants per tray, 23.3 cm × 16.0 cm × 6.0 cm) according to Reboud et al.^28^. The experiment was conducted with three replications and repeat twice. The aboveground shoots of each plant were harvested at 50 DAT and the leaf area and the dry weight were measured. RCC was calculated according to the formula: RCC = ({(DB_S_^1:3^/DB_R_^1:3^) + (DB_S_^1:1^/DB_R_^1:1^) + (DB_S_^3:1^/DB_R_^3:1^)}/N)/(DB_S_^1:0^/DB_R_^1:0^)^29,30^. DB_S_^n:n^ and DB_R_^n:n^ means the dry weight or the leaf area of each plant in susceptible and resistant subpopulations planted at ratio of n:n. N is the number of mixed ratio; here N = 3. RCC value greater than 1.0 suggested a superior competition ability of susceptible population. While RCC value less than 1.0 indicated lower competition ability of susceptible population.

### Statistical analysis

The data of bioassay was analyzed with SigmaPlot 12.0 (Systat Software, San Jose, CA). The statistical difference of the leaf area, dry weight, RGR, LAR, NAR of *D. sophia* populations with different ALS mutation were subjected to one-way analysis of variation (ANOVA) followed by Tukey’s multiple comparisons test using SPSS 16.0 (SPSS, Chicago, IL, USA). The criterion for statistical significance was *P* < 0.05.

### Ethics declarations

*Descurainia sophia* is a notorious weed widely distributed in the wheat fields or wild field of Henan and Xinjiang Province in China. Farmers need to spend a lot of money and manpower to get rid of them in their fields each year. In fact, common weed seeds collection in China is allowed and frequently used in weed research. Thus, collection of *D. sophia* seeds is permitted naturally and had no influence on the environment. Besides, we have got the oral permission of the field owners when *D. sophia* seeds were collected from wheat fields.

## Supplementary Information

Supplementary Information.

## Data Availability

The datasets generated during and/or analyzed during the current study are available from the corresponding author on reasonable request.
