# Supplementary material for: Characterization of resistance and fitness cost of Descurainia sophia L. populations from Henan and Xinjiang, China
Source: Sci Rep. 2021 Jul 19;11:14655. doi: 10.1038/s41598-021-94317-y (PMC8290039; doi:10.1038/s41598-021-94317-y)
Supplement: Supplementary file 1 — Supplementary Information. [file 41598_2021_94317_MOESM1_ESM.pdf]

Characterization of resistance and fitness cost of *Descurainia sophia* L. populations from Henan and Xinjiang, China

Dongzhi Li[a], Lanfen Xie[a], Pei Zhang[a], Runqiang Liu[a], Mingwang Shi[a], Yu Mei[b,c] *, Li Xu[a,] *

[a] *College of Resources and Environment, Henan Institute of Science and Technology, Xinxiang, 453003, Henan Province, China.*

[b] *State Key Laboratory of Desert and Oasis Ecology, Xinjiang Institute of Ecology and Geography, Chinese Academy of Sciences, Urumqi, 830011, China.*

[c]*The Specimen Museum of Xinjiang Institute of Ecology and Geography, Chinese Academy of Sciences,Urumqi 830011, China.*

***Corresponding author:**

Dr. Yu Mei, Address: Beijing South Road, Xinshi District, Xinjiang Institute of Ecology and Geography, E-mail: meiyu1018@126.com;

Dr. Li Xu, Address: Hualan Road, Hongqi District, Henan Institute of Science and Technology, E-mail: xuli-apple-love@163.com.

Table S1. Geographical information of *D. sophia* populations collected from Henan and Xinjiang

Population	Date	Location	Longitude (°E)	Latitude (°N)
H1	2017.5	Xinxiang	35.35110	114.02222
H2	2017.5	Jiaozuo	35.08914	113.18879
H3	2017.5	Zhengzhou	34.56041	113.67615
H4	2017.5	Pingdingshan	33.84140	112.96173
H5	2017.5	Kaifeng	34.83105	114.53652
H6	2017.5	Xuchang	33.86552	113.70118
H7	2017.5	Xuchang	34.19133	114.09114
H8	2017.5	Shangqiu	34.22530	115.23461
H9	2017.5	Shangqiu	34.54991	115.88262
H10	2017.5	Shangqiu	34.14413	116.23873
H11	2017.5	Zhoukou	33.31003	114.47851
X1	2015.7	Shihezi	44.41525	86.15200
X2	2015.7	Tarbagatay	44.79928	85.11283
X3	2015.7	Changji	44.17731	86.55542
X4	2015.7	Changji	44.17850	86.71003
X5	2015.7	Changji	44.15561	86.85547
X6	2015.7	Changji	43.99972	87.07742
X7	2015.7	Tarbagatay	46.77611	83.82992
X8	2015.7	Tarbagatay	46.77765	83.61775
X9	2015.7	Tarbagatay	46.70042	82.83166
X10	2015.7	Bortala	44.90600	82.32117
X11	2015.7	Ili	43.79761	81.54097
X12	2015.7	Ili	43.48225	82.26128
X13	2015.7	Urumqi	43.86572	87.57006
X14	2015.7	Shihezi	44.27581	86.03092
X15	2015.7	Changji	43.97578	89.74878
X16	2015.7	Changji	43.98200	89.74700
X17	2015.7	Changji	44.01375	89.73289
X18	2015.7	Altay	47.02247	89.75483
X19	2015.7	Altay	47.20867	89.80375
X20	2015.7	Altay	48.70592	86.75411
X21	2016.5	Kashgar	38.23981	77.30361
X22	2016.5	Kashgar	37.83364	77.45558
X23	2016.5	Kashgar	37.97742	77.45436
X24	2016.5	Kashgar	38.12678	77.38689
X25	2016.5	Kashgar	38.48606	77.18453
X26	2016.5	Kashgar	38.89189	77.53928
X27	2016.5	Kashgar	38.17677	77.26563
X28	2016.5	Kashgar	39.36872	76.03311
X29	2016.5	Kashgar	39.32483	76.12600
X30	2016.5	Kashgar	39.18261	76.17122
X31	2016.5	Kashgar	39.40133	75.88025

Table S2 ALS-inhibiting herbicides used for dose-response and cross-resistance determination in *D. sophia*

Herbicides	Group	Fomulation	Company	Recommend dose (g ai ha^{-1})
tribenuron-methyl	SU	75% WG	Shandong Shenbon Greenland Chemical	18.0
flucarbazone-sodium	SCT	75% WG	Shandong Binnong Technology	45.0
bensulfuron-methyl	SU	32% WP	Jiangsu Kuaida Agrochemical	65.7
flumetsulam	TP	80% WG	Dow AgroSciences	30.0
florasulam	TP	50g/L SC	Shandong Huiminzhonglian	4.5
pyroxsulam	TP	7.5% WG	Dow AgroSciences	14.1
imazapic	IMI	240 g/L AS	BASF	108.0
bispyribac-sodium	PTB	10% SC	Anhui Xingyu	45.0

Table S3. The leaf area and dry weight of *D. sophia* subpopulations from Henan and Xinjiang under monoculture condition

Population	Leaf area (cm^2 per plant)			Dry weight（mg per plant）		
	28 DAT	35 DAT	42 DAT	28 DAT	35 DAT	42 DAT
SX13	6.13±1.54ab	10.07±1.40b	22.31±2.32b	9.8±2.1abc	22.4±4.3b	53.1±5.1bc
SX30	4.79±1.12a	5.70±0.88a	28.82±2.67c	6.2±1.1a	10.6±4.1a	69.2±6.5d
SX31	5.03±0.64a	13.27±1.33c	31.73±2.56c	6.7±0.7ab	25.4±7.5b	68.9±5.8d
SH5	6.40±1.56ab	11.49±1.93bc	13.89±1.63a	10.5±1.7bc	24.4±5.3b	50.6±4.8b
SH6	3.72±0.63a	6.74±0.68a	20.90±1.78b	5.9±1.7a	10.2±0.8a	41.6±4.2a
SH7	8.77±1.53b	10.85±1.35bc	30.87±2.89c	12.4±2.7c	20.7±1.2b	59.2±5.7c

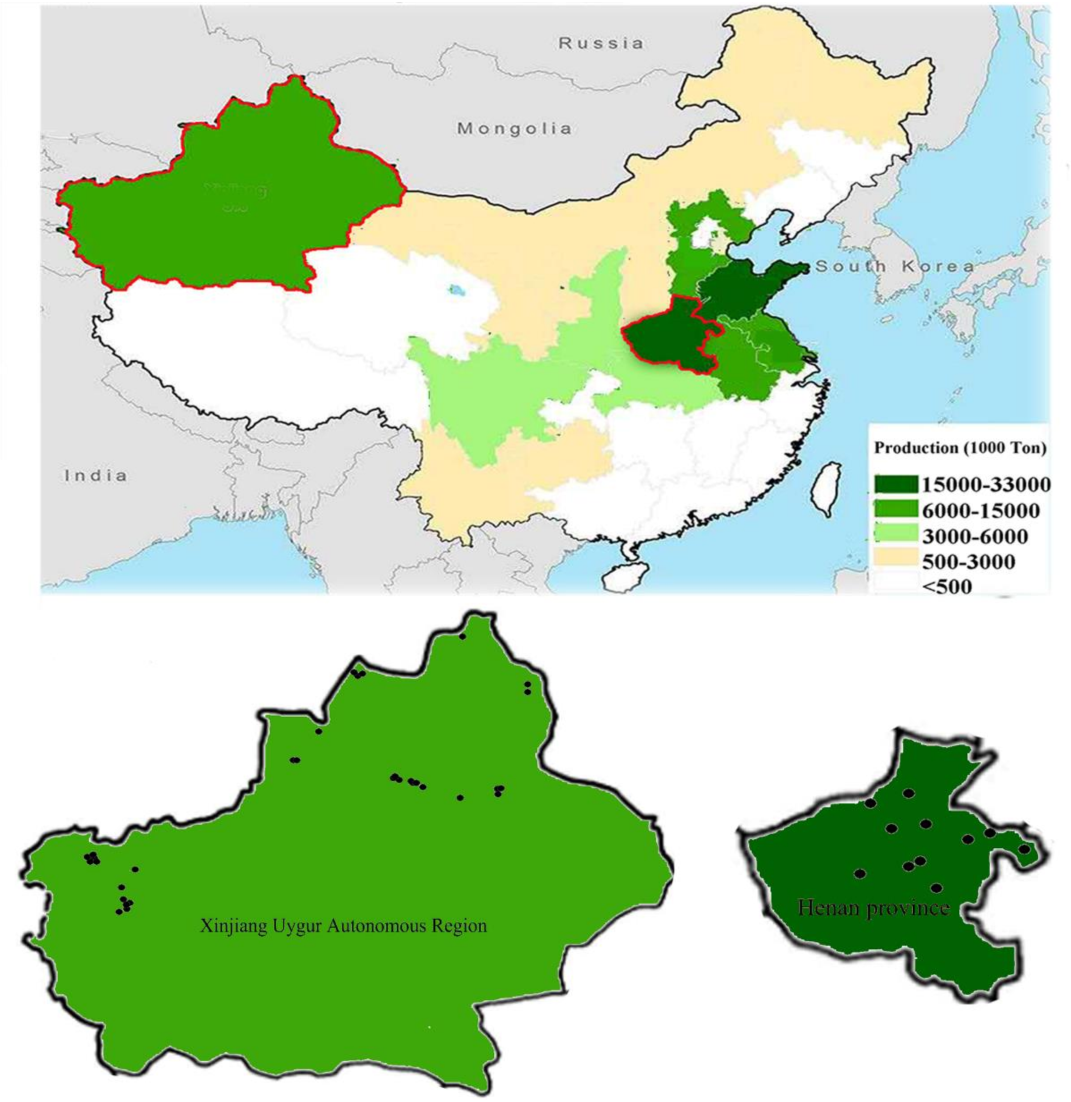

Figure S1. Distribution of *D. sophia* populations collected from Henan and Xinjiang and the main wheat production area in China. The production data was cited from National Bureau of Statistics of China (https://mp.weixin.qq.com/s/x7pO3wqijJ8dtrvz5mTS0A).
